# Automatic extraction of 2D patellofemoral bone morphology and alignment measures from 3D models in a young adolescent population

**DOI:** 10.1016/j.ostima.2026.100394

**Published:** 2026-05-12

**Authors:** Rosemarijn van Paassen, Nazli Tümer, Inge Bosch, Jukka Hirvasniemi, Erin M. Macri, Eva Langius, Amy Roos, Tom M. Piscaer, Amir A. Zadpoor, Stefan Klein, Sita M.A. Bierma-Zeinstra, Edwin H.G. Oei, Marienke van Middelkoop

**Affiliations:** a Erasmus Medical Center Rotterdam, Dr. Molewaterplein 40, 3015 GD Rotterdam; b Technical University Delft, Mekelweg 5, 2628 CD Delft

**Keywords:** Patella, Femur, Knee, Concurrent validity, Alignment variables, Morphology measures, Automatic extraction

## Abstract

**Objective:**

To determine the concurrent validity of automatically extracted alignment variables and morphology measures and to establish reference values and evaluate sex-based differences among a young adolescent population.

**Design:**

Data from 1912 participants who underwent knee MRI within a large prospective population cohort study were included. Patellar and femoral landmarks were automatically extracted from two previously created statistical shape models (SSMs). Landmarks were extracted manually in 2D, manually in 3D and using the automated method of landmark extraction in 3D. Using these landmarks, four patellofemoral alignment variables and thirteen morphology measures were calculated. Concurrent validity of alignment variables and morphology measures calculated using automatically extracted landmarks compared to manual annotation on MRI was tested using a two-way, mixed-effects model with multiple raters and absolute agreement ICC. Differences between boys and girls were determined using an independent samples two-tailed *t*-test.

**Results:**

Two alignment variables and seven morphology measures demonstrated good validity with the automated SSM-based method, with an ICC>0.75 for patellar tilt angle (ICC=0.83), bisect offset (ICC=0.93), epicondylar width (ICC=0.95), patellar width (ICC=0.86), patellar length (ICC=0.83), femoral notch width (ICC=0.90), femoral notch depth (ICC=0.83) medial (ICC=0.94) and lateral condyle length to epicondylar width ratio (ICC=0.92). Reference values of the two alignment and seven morphology measures all differed between boys and girls.

**Conclusion:**

Alignment variables and morphology measures can be accurately determined in 3D. All alignment variables and morphology measures, with at least a good ICC, differed between boys and girls.

## Introduction

Normal knee morphology and alignment contribute to optimal knee function, as both play an important role in distributing weight and stress across the knee joint [1]. Alignment is influenced by bone shape and the relative positioning of bones. To evaluate patellofemoral alignment and morphology in 2D, alignment variables, such as the patellar translation measure, are often used [2,3].

Current research on patellofemoral alignment variables is mainly performed in adult and disease-specific populations. For example, it is known that deviations of several alignment variables are associated with the presence of patellofemoral pain (PFP) [4,5], patellofemoral instability [6,7], and patellofemoral osteoarthritis (PFOA) [2]. Moreover, abnormal patellar alignment is more common in patients that undergo anterior cruciate ligament reconstruction [8]. In children and adolescents, patellofemoral alignment variables have been primarily investigated in populations with specific musculoskeletal disorders (e.g. Osgood-Schlatter (OSD), Sinding-Larsen-Johansson syndrome (SLJS) or PFP) [9], without knowledge of reference values for alignment variables in young-adolescents.

Patellofemoral alignment variables are typically calculated by identifying various bony landmarks using radiography, computed tomography (CT), or magnetic resonance imaging (MRI) [10]. The coordinates of these landmarks are needed to calculate distances, ratios, and angles, yielding information on bone morphology and alignment. In the current clinical setting, the landmarks are often determined manually by identifying them on radiography or a pre-specified CT or MRI slice. While studies have demonstrated adequate intra- and inter-rater reliability in adult populations [11,12], the process remains time-consuming and may still be subject to variability [13]. Moreover, positioning of patients during imaging, slice selection on MRI/CT, and annotation of landmarks on bone or cartilage all influence the accuracy of the calculated 2D alignment variables. Furthermore, different methods of annotation used in studies can lead to variations in the results. For instance, femoral landmarks are either annotated on the most proximal slice that shows full cartilage coverage of the trochlear groove in a medial-to-lateral direction or on the slice showing the largest posterior condyles [10,12,14]. This introduces discrepancies between the methods. Automating the extraction of these landmarks in 3D may help to overcome these issues.

Statistical shape modeling (SSM) has been increasingly used in bone anatomy research. SSM is a method that describes the mean and variation of shape within a study population. A principle called corresponding points is used to create the SSM, ensuring the anatomical consistency of the points describing bone shape across all (3D) bone models used for creating the SSM [15]. In this process, the number of points doubles with each iteration, and their locations are optimized to ensure anatomical correspondence across all shapes. This process in creating the SSM enables the automatic extraction of bony landmarks, thereby eliminating the time and consistency limitations of the manual method described above.

Therefore, to be able to examine whether widely used 2D measures can be meaningfully translated to 3D measures, the primary aim of this study is to determine the concurrent validity of the alignment variables calculated using automatically-extracted 3D landmarks in young adolescents. Second we will determine reference values for these 3D alignment variables and morphology measures in adolescents and explore differences between boys and girls. Additionally, intra-rater reliability between twice manually annotated landmarks on 2D MRI slices and 3D models, as well as concurrent validity of manual annotation on 3D models, will be determined.

## Methods

### Study sample

A subgroup of participants from the Generation R study was included in the present study. Generation R is a large population-based cohort study that follows children and their parents from fetal life until adulthood. In total, 9778 pregnant women and their children born between 2002 and 2006 were included in the cohort study [16]. At multiple time points (*i.e*., 5, 9, and 13 years old), the children and their parents visited the research center for physical measurements, questionnaires, and imaging. The Medical Ethics Committee of Erasmus Medical Center (Rotterdam, The Netherlands) approved all measurements within the Generation R study. At a follow-up moment at age 13, a random subset of 1912 participants underwent an MRI of both knees. All participants who underwent these knee MRIs were included in the current study.

### Measurements: questionnaires, MRI and related measurements

Questionnaire data extracted for the current study included age, sex, and ethnicity. From physical measurements, the children’s height (measured using a Harpenden stadiometer (Holtain Limited, Crymych, United Kingdom)) and weight (measured without heavy clothing and shoes using a mechanical scale (SECA, Hamburg, Germany)) were used to calculate body mass index and age- and sex-adjusted standard deviation scores (BMI-z) in accordance with the Dutch reference charts [17]. A knee MRI was performed using a 3.0T MRI scanner (Discovery MR750w, GE Healthcare, Milwaukee, WI, USA). Imaging was performed in supine position with two knees fully extended within the coil. No further standardization steps were performed. The scanning protocol included a 3D water excitation Gradient Recalled Acquisition in Steady State (3DGRASS) sequence, acquired in the coronal plane and reconstructed in the sagittal and axial planes.

### Patellofemoral alignment variables and morphology measurements

**Fig. 1:**
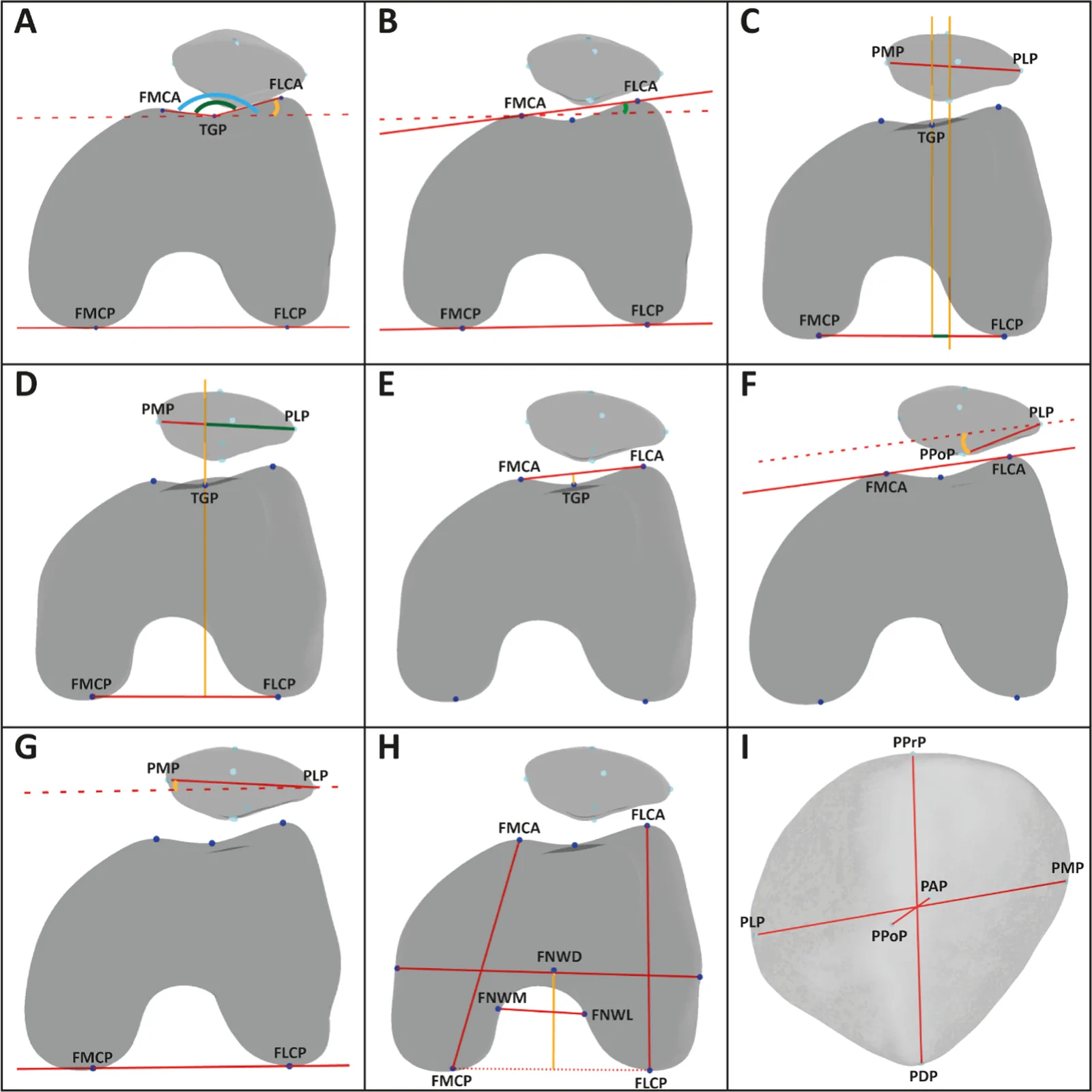
KAPs: The red lines are lines between 2 points. The red dashed lines are reference lines. The orange lines are orthogonal to the red lines. PCL = posterior condyle line, TGP = trochlear groove deepest point, ACL = anterior condyle line. (A) Lateral trochlear inclination: angle between the lines through the lateral anterior condyle and TGP and the PCL (in green) & Medial trochlear inclination: angle between the line through the medial anterior condyle and TGP and the PCL (in light blue), and Sulcus angle: angle between the condylar outsets (in yellow). (B) Trochlear angle: angle between the ACL and PCL. (C) Patellar lateral translation: Distance between two lines perpendicular to the PCL through the deepest point of the trochlea and most posterior point of the patella. (D) Bisect offset: Percentage of patellar width lateral from the orthogonal line from the PCL through the TGP. (E) Sulcus depth: Distance between the ACL and TGP. (F) Lateral patellar tilt angle: Angle between the ACL and the line connecting the most lateral and most posterior patella points. (G) Patellar tilt angle: Angle between the PCL and the line connecting the most medial and lateral patella points. (H) Femoral morphology: epicondyle width, lateral/medial condyle height/length, notch depth and width. (I) Patellar morphology: width/length/thickness of the patella.

The following four patellofemoral alignment variables were extracted: patellar tilt angle, lateral patellar tilt angle, patellar lateral translation, and bisect offset. Additionally, thirteen specific morphology measures were extracted: sulcus angle, sulcus depth, trochlear angle, and medial/lateral inclination, patellar width/length/thickness, femoral notch width/depth, epicondylar width, and the ratio between epicondylar width and medial/lateral length (anterior-posterior (AP)). Landmarks needed to calculate alignment variables and morphology measures included seven patellar and eleven femoral landmarks, indicating the most proximal, distal, medial, lateral, anterior, and posterior locations on both bone surfaces. Fig. 1 and Table 1, Table 2 present an overview of all the alignment variables, morphology measures, and the landmarks and their corresponding definitions [3,12].

**Table 1:** Overview of landmarks on the patella and the femur used to calculate alignment variables.

Patella			Femur		
Landmarks	**Abbreviation**	**Definition**	**Landmarks**	**Abbreviation**	**Definition**
Patella proximal pole	PPrP	Most proximal point of the patella	**Trochlear groove deepest point**	TGP	Deepest point of the trochlear groove in an axial view
Patella distal pole	PDP	Most distal point of the patella	**Femoral medial condyle anterior**	FMCA	Most anterior point of the medial condyle
Patella anterior point	PAP	Most anterior point of the patella	**Femoral medial condyle anterior**	FLCA	Most anterior point of the lateral condyle
Patella medial point	PMP	Most medial point of the patella	**Femoral medial condyle posterior**	FMCP	Most posterior point of the medial femoral condyle
Patella lateral point	PLP	Most lateral point of the patella	**Femoral lateral condyle posterior**	FLCP	Most posterior point of the lateral femoral condyle
Patella posterior point	PPoP	Most posterior point of the patella	**Femoral medial epicondyle**	FMCE	Most medial point of the medial condyle
			**Femoral lateral epicondyle**	FLCE	Most lateral point of the lateral condyle
			**Femoral notch width medial**	FNWM	Most lateral point of the medial condyle
			**Femoral notch width lateral**	FNWL	Most medial point of the lateral condyle
			**Femoral notch width deepest**	FNWD	Deepest point of the posterior notch

**Table 2:** Overview of the knee alignment variables and morphology measures of interest and the landmarks needed to calculate all variables and morphology measures.

Alignment variables and morphology measures	Definition	Landmarks needed
Lateral trochlear inclination (Fig. 1A)	The angle between the lines through the deepest point of the trochlear groove and the most anterior point of the lateral condyle and the posterior condyle line.	TGP, FLCA, FMCP, FLCP
Medial trochlear inclination (Fig. 1A)	The angle between the lines through the deepest point of the trochlear groove and the most anterior point of the medial condyle and the posterior condyle line.	TGP, FMCA, FMCP, FLCP
Sulcus angle (Fig. 1A)	Angle between the lines from the medial anterior condyle and the lateral anterior condyle to the deepest point of the trochlear groove	TGP, FMCA, FLCA
Sulcus depth (Fig. 1E)	Depth of the sulcus groove as compared to the anterior condyles	TGP, FMCA, FLCA
Lateral patellar tilt angle (Fig. 1F)	The angle formed between the line drawn parallel to the lateral patellar facet and the line drawn connecting the most anterior points of the medial and lateral condyles	FMCA, FLCA, PLP, PPoP
Patellar lateral translation (Fig. 1C)	Distance between two orthogonal lines on the posterior condyle line. One line through the deepest point of the trochlea and one line through the most posterior point of the patella	PPoP, TGP, FMCP, FLCP
Trochlear angle (Fig. 1B)	The angle between the line through the anterior condyles and the line through the posterior condyles	FMCA, FLCA, FMCP, FLCP
Patellar tilt angle (Fig. 1G)	The angle between the line through the greatest width of the patella and the line through the posterior condyles	PMP, PLP, FMCP,FLCP
Epicondylar width (Fig. 1H)	Distance between the most medial and most lateral point of the femur	FMCE, FLCE
Bisect offset (Fig. 1D)	Percentage of the width of the patella that lies lateral to the line orthogonal to the posterior condyle line and through the trochlear groove	PMP, PLP, TGP, FMCP, FLCP
Lateral condyle length:epicondylar width ratio (Fig. 1H)	Ratio between lateral condyle length in the AP view and the epidondylar width	FLCA, FLCP, FMCE, FLCE
Medial condyle length:epicondylar width ratio (Fig. 1H)	Ratio between the medial condyle length in the AP view and the epicondylar width	FMCA, FMCP, FMCE, FMCL
Patellar width (Fig. 1I)	Width of the patella	PMP, PLP
Patellar length (Fig. 1I)	Length of the patella	PPrP, PDP
Patellar thickness (Fig. 1I)	Thickness of the patella	PAP, PPoP
Femoral notch width (Fig. 1H)	Inner width of the posterior femoral notch	FNWM, FNWL
Femoral notch depth (Fig. 1H)	Depth of the posterior femoral notch calculated as the distance of the deepest point of the femoral notch to the posterior condyle line	FNWD, FMCP, FLCP

AP=anterior – posterior.

FMCA, the most anterior point on the medial femoral condyle; FLCA, the most anterior point on the lateral femoral condyle; FMCP, the most posterior point on the medial femoral condyle; FLCP, the most posterior point on the lateral femoral condyle; FNWD, the deepest point of the posterior notch; FNMW, the most medial point of the femoral notch; and FNLW, the most lateral point of the femoral notch.

Landmarks were extracted in three different ways. The landmarks were manually annotated on 2D MRI slices and 3D models from the left knee of 30 randomly selected participants (15 boys and 15 girls). Furthermore, the landmarks were automatically extracted from all available 3D models as well.

### Manual annotation of landmarks on MRI

Manual (2D) annotation of the landmarks was performed using 3D Slicer (version 5.6.2), based on the method of Macri et al. (2017) [12]. First, the axial MRI slice showing the largest area of the posterior femoral condyles was selected. The deepest point of the trochlear groove (TGP), the most anterior point on the medial femoral condyle (FMCA), the most anterior point on the lateral femoral condyle (FLCA), the most posterior point on the medial femoral condyle (FMCP), the most posterior point on the lateral femoral condyle (FLCA), the deepest point of the posterior notch (FNWD), the most medial point of the femoral notch (FNMW), and the most lateral point of the femoral notch (FNLW) were annotated using this slice. Then, the axial slice with the largest epicondylar width was selected to annotate the most medial and lateral points of the femoral epicondyle (FMCE and FLCE). Two patellar points were annotated on the sagittal slice that shows the largest patella surface. The most proximal point of the patella (PPrP) and the most distal point of the patella (PDP) were annotated using this slice. Four additional patella points were annotated in the axial slice that shows the largest oblique distance. These were the most medial (PMP), lateral (PLP), anterior (PAP), and posterior (PPoP) points. Manual annotation was performed twice, a week apart in a random order, on all MRIs by one researcher (R.P.).

### Automated extraction of 3D landmarks from the SSM

Previously, two 3D SSMs of the patella and femur were created using all knee MRIs of the 13-year-old participants in the Generation R cohort [18]. In short, an automatic segmentation algorithm consisting of a spatial (multi-atlas) and an appearance (random forest classifier) components was trained and validated using 30 manually segmented MRIs. This algorithm was then used to segment all the remaining MRIs within the study. After visual inspection, manual correction, and post-processing (including smoothing and remeshing), a dataset of 3D models of 3638 patellae and 3355 femurs was produced. These 3D models were used to create an SSM of the patella and the femur separately [18]. All the 3D models were aligned using a unbiased registration algorithm and correspondence was established across the aligned samples using a closest point algorithm [15]. The landmarks of interest were manually annotated on the mean shape in the in-house built viewer in MATLAB (MATLAB 2023b, Mathworks, Massachusetts, USA), and the corresponding points, and therefore all landmarks were automatically extracted from all other 3D bone models. All landmarks were annotated once on the mean patellar and mean femoral 3D shapes by one researcher (R.P.) and visually inspected by another researcher (N.T.).

Most landmarks in 3D space are located in approximately the same plane as would be expected on a 2D MRI slice. For example, the position of the most anterior point of the lateral femoral condyle is not expected to change much between 2D and 3D annotations. There are, however, a few landmarks (such as the PAP and TGP) that we would expect to fall outside of a 2D plane and thus potentially resulting in different estimates of alignment.

### Manual annotation of landmarks on 3D models

All landmarks were manually annotated using 3D Slicer (version 5.6.2) on the previously created 3D models of the left knees of the same 30 randomly selected participants, who were included in manual annotation on MRI. Annotation in 3D was done as follows: all femur landmarks were annotated in the distal view. Afterwards, the view was rotated posteriorly and then anteriorly, and the landmarks were adjusted if necessary. Patellar landmarks PMP, PLP, PPrP, and PDP were annotated in the posterior view. Then, when rotating to a superior view, the PAP and PPoP were annotated. The PDRP was then finally annotated in a side/medial-lateral view. Manual annotation on all 30 3D models was performed twice, a week apart in a random order, by one researcher (R.P.).

### Calculation of alignment variables

**Fig. 2:**
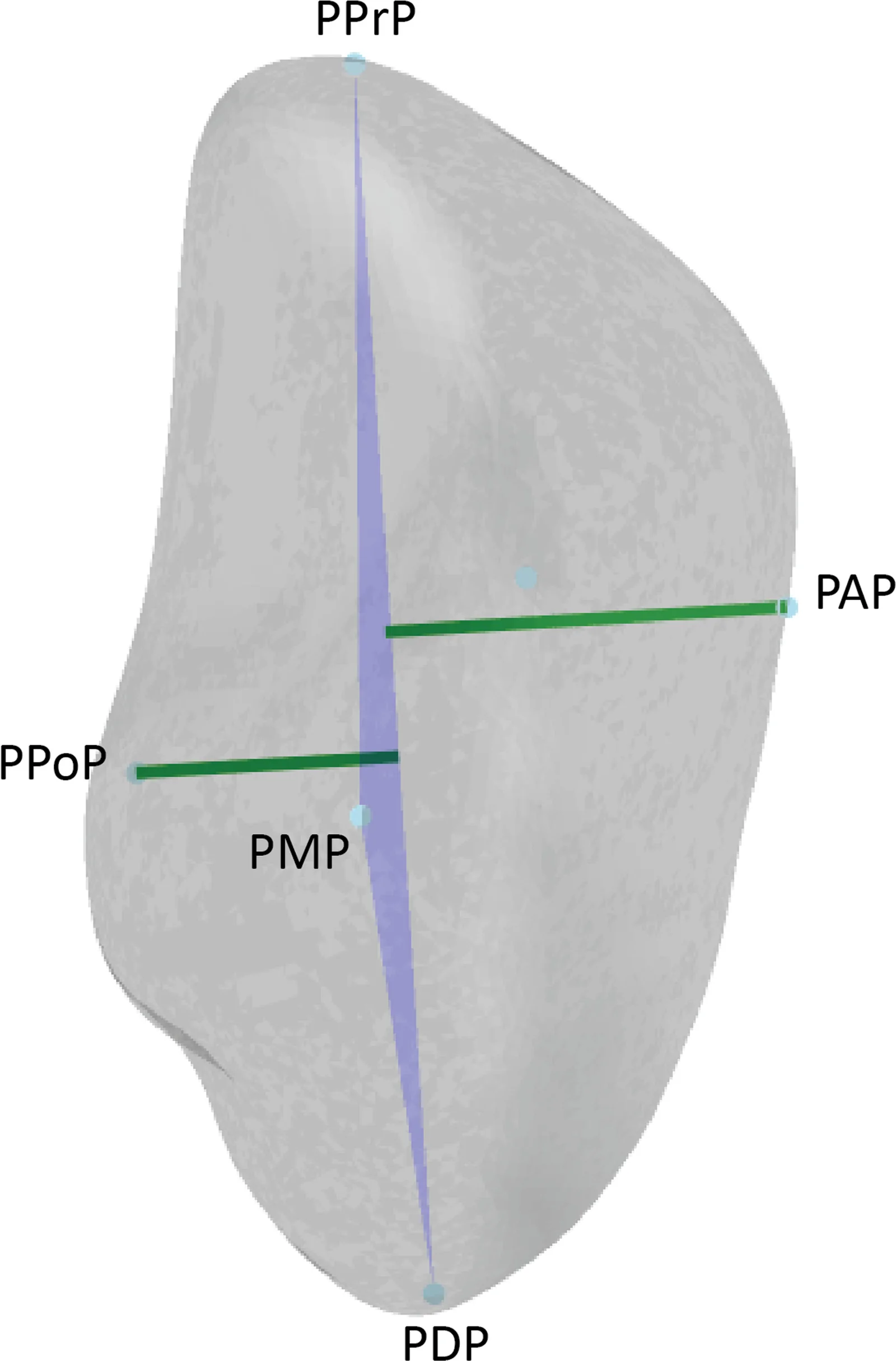
A sagittal view of the patella with an example of the calculation of patellar thickness in 3D. The sum of the length of the two green lines was taken as a representation of patellar thickness instead of the distance between PAP and PPoP itself. Abbreviations: PPoP = patella posterior point, PAP = patella anterior point, PMP = patella medial point, PDP = patella distal point, PPrP = patella proximal point.

Calculations were performed using Python (version 3.12.8, Python Software Foundation, https://python.org). Except for patellar thickness, the same formulas were used to calculate distances and angles from landmarks extracted manually in 2D, manually in 3D, and using the automated method of landmark extraction in 3D. Since 2D manual landmarks were annotated on the same plane, the z-coordinate was identical for all landmarks. Patellar width, length, femoral notch width, and epicondylar width were calculated using Euclidean distances between two landmarks. Patellar thickness was also calculated as the distance between two points (the most anterior and most posterior points of the patella) using the 2D manually annotated landmarks. The line between PAP and PPoP was projected on the normal of the plane spanned by PPrP, PMP, and PDP to calculate the patellar thickness in 3D. These individual distances were then summed to obtain the overall deviation of the PAP and PPoP to the reference plane (Fig. 2). A different calculation method was employed in 3D than in 2D because the most anterior and most posterior points of the patella were not necessarily positioned at the same height in 3D, resulting in an overestimation when calculating the Euclidean distance in 3D.

All landmarks were annotated twice for manual annotation on the 30 2D MRI slices and the 3D models, and the alignment variables and morphology measures calculated using these manual landmarks were then averaged to minimize intrarater variability.

### Statistical analysis

Descriptive statistics were used to describe the characteristics of the study participants. Intraclass correlation coefficients (ICCs) and the standard error of measurements (SEMs, calculated as SD × √(1 − ICC)) were used to determine concurrent validity and both were reported with their 95% confidence intervals (CIs) [19]. The concurrent validity between alignment variables and morphology measures calculated using manually annotated (gold standard) landmarks on MRI and automatically extracted landmarks was tested using a two-way, mixed-effects model with multiple raters and absolute agreement ICC (3,k) [20]. ICCs were calculated between the average of the two manual annotations and the automatic annotations, and SEMs, bias, and limits of agreement were also extracted. In addition to our primary research question, we determined test-retest intra-rater reliability between the alignment variables and morphology measures calculated with the two manual annotations on 2D MRI slices and the 3D models using a two-way mixed-effects model with a single rater and absolute agreement ICC(3,1). We also determined concurrent validity between the average of the two manual annotations on the 3D models and the automatically extracted landmarks using a two-way mixed-effects model with multiple raters and absolute agreement ICC (3,k).

Additionally, the agreement between the alignment variables and morphology measures calculated using manually annotated and automatically extracted landmarks was visualized using Bland-Altman plots. Bland-Altman plots were also used to ensure homoscedasticity and to evaluate if any systematic bias occurred. ICCs were deemed poor (0.50), moderate (0.50–0.75), good (0.75–0.90), or excellent (>0.90) [6,21]. A lower ICC may in some cases still be clinically useful, depending on the reason for taking the measure and how it will influence clinical decision making. For the alignment and morphology measurements that showed a good or excellent agreement (ICC > 0.75), reference values for the study population were determined using the automatically extracted landmarks from all 3D models included in the SSMs. The mean, standard deviation, 2.5 and 97.5 centiles of each variable were calculated to generate reference values for the alignment variables and morphology measures. Additionally, differences in these variable values between boys and girls were determined using a two-tailed independent samples *t*-test with a threshold for significance of *p* < 0.05. Statistical analyzes were performed using R statistical software (version 4.3.2; R Core Team, 2021). Bland-Altman plots were created using the ggplot2 package, and ICCs were calculated using the irr package in R.

## Results

This study included data from 1912 participants who underwent MRI of the knee. After checking all segmentations, 3638 patella and 3355 femur 3D models from 3691 different knees of 1886 participants were included in the SSM. Participants had a mean age of 14.1 (SD 0.7) years during MRI, with 52.5% of knees being from girls. Participants had a mean (SD) BMI-z score of 0.4 (1.2) kg/m^2^with a mean height and weight of 164.4 (7.9) cm and 53.7 (11.0) kg. Most participants had Dutch ethnicity (60%), followed by other Western (29%) and non-Western (8%).

**Fig. 3:**
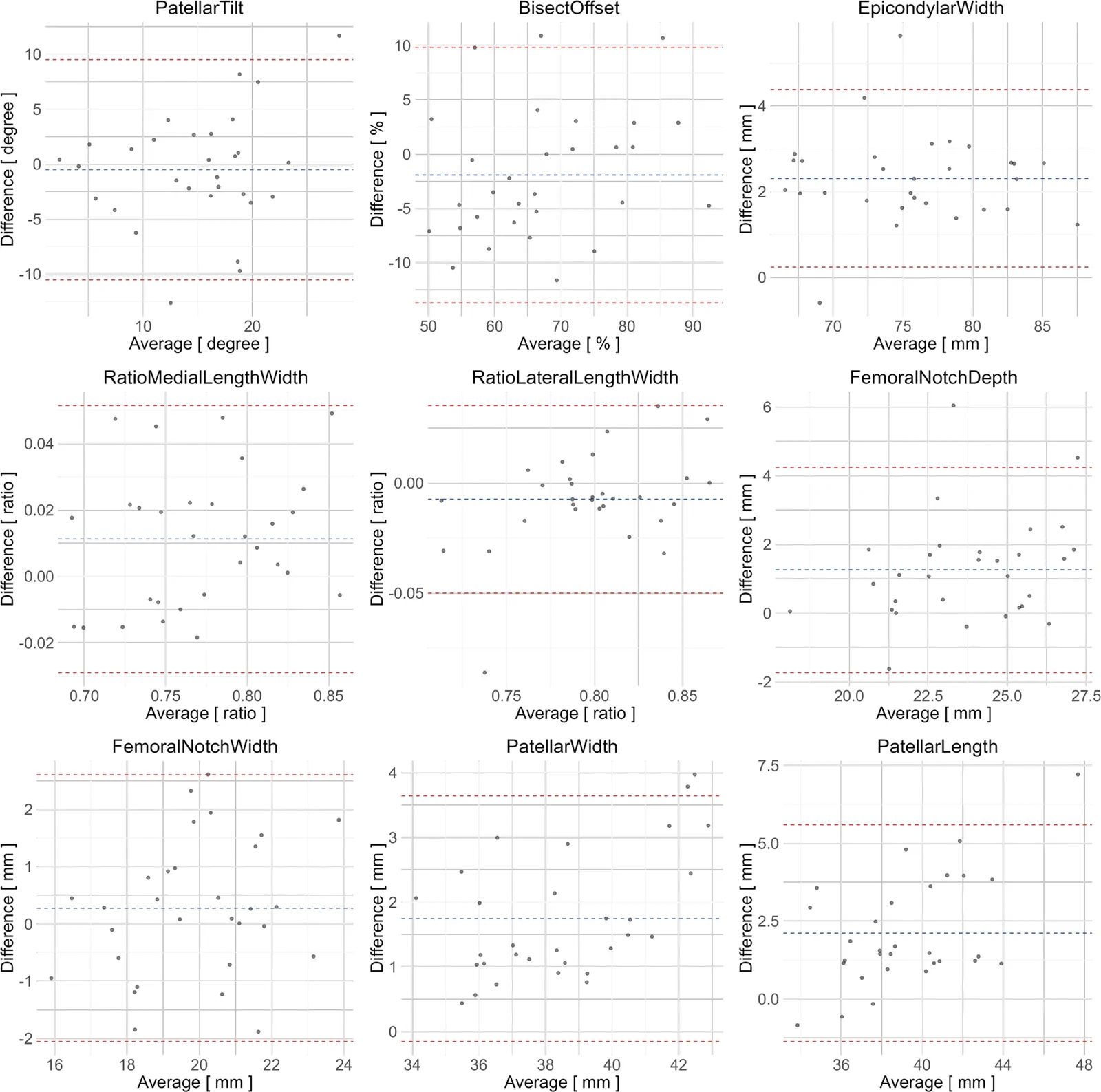
Bland-Altman scatterplots for the nine alignment variables and morphology measures with an ICC>0.75. Showing agreement between calculating variables using automatically extracted landmarks and the mean of the two manual annotated landmarks. Epicondylar width, femoral notch width and patellar width are expressed in millimeters, while the lateral and medial condyle length to width and bisect offset are expressed in ratios.

Concurrent validity ICC values between manual annotation on 2D MRI slices and automatic extraction ranged from 0 to 0.95, with the lowest value corresponding to the trochlear angle and the highest to the epicondylar width (Table 3). SEMs ranged from 0.01 for lateral condyle length to epicondylar width ratio to 4.40 for the sulcus angle, except for the medial inclination angle with an SEM of 5.71. Bland-Altman plots of the alignment variables and morphology measures with an ICC>0.75 showed no signs of heteroscedasticity, but did show systematic bias for epicondylar width (2.31 mm), femoral notch depth (mean bias of 1.26 mm), and patellar width (mean bias 1.75 mm) and length (2.11 mm) (Fig. 3 and Table 3). There were two alignment variables and seven morphology measures with an ICC > 0.75 that were analyzed further for determining reference values (Table 3).

**Table 3:** Inter-method concurrent validity of alignment variables and morphology measures calculated with automatically extracted landmarks.

Alignment variable or morphology measure	ICC [95% CI]	SEM [95% CI]	Average (mean value)	Bias (mean difference)	Limits of agreement
Patellar tilt angle [degrees]	**0.83 [0.63; 0.92]**	2.13 [1.47; 3.09]	14.89	−0.51	−10.54; 9.51
Lateral patellar tilt angle [degrees]	0.62 [0.22; 0.82]	3.43 [2.38; 4.93]	10.29	−2.04	−13.00; 8.92
Lateral patellar translation [mm]	0.21 [−0.08; 0.61]	2.60 [1.84; 3.04]	14.95	10.04	4.31; 15.77
Bisect offset %	**0.93 [0.84; 0.96]**	1.64 [1.13; 2.40]	67.18	−1.94	−13.71; 9.82
Sulcus angle [degrees]	0.16 [−0.07; 0.53]	4.40 [3.30; 4.96]	152.02	−17.80	−27.2; −8.39
Sulcus depth [mm]	0.14 [−0.08; 0.49]	0.78 [0.60; 0.87]	3.96	2.93	1.28; 4.58
Trochlear angle [degrees]	0 [−0.10; 0.17]	4.33 [3.95; 4.55]	8.94	−9.17	−17.67; −0.68
Inclination angle[degrees] Lateral Medial	0.54 [−0.14; 0.8] 0.28 [−0.49; 0.65]	2.36 [1.55; 3.72] 5.71 [3.95; 4.55]	17.52 162.73	2.72 −1.44	−4.11; 9.54 −14.63; 11.75
Epicondylar width [mm]	**0.95 [−0.01; 0.99]**	0.23 [0.11; 1.06]	75.71	2.31	0.24; 4.38
Condyle length (AP) to epicondylar width ratio Lateral Medial	**0.92 [0.83; 0.96]** **0.94 [0.83; 0.97]**	0.01 [0; 0.01] 0.01 [0; 0.01]	0.80 0.77	−0.01 0.01	−0.05; 0.04 −0.03; 0.05
Patellar [mm] Length Thickness Width	**0.83 [−0.04; 0.95]** 0.73 [0.00; 0.90] **0.86 [−0.16; 0.96]**	0.74 [0.40; 1.81] 0.64 [0.39; 1.24] 0.37 [0.18; 1.04]	39.23 19.09 38.48	2.11 −1.14 1.75	−1.37; 5.60 −3.57; 1.29 −0.15; 3.64
Femoral notch width [mm]	**0.90 [0.80; 0.95]**	0.37 [0.26; 0.53]	19.89	0.27	−2.05; 2.60
Femoral notch depth [mm]	**0.83 [0.33; 0.94]**	0.63 [0.38; 1.25]	23.74	1.26	−1.73; 4.24

An ICC between 0.5 and 0.75 is deemed moderate, between 0.75 and 0.9 good and above 0.9 excellent. ICCs above 0.75 are shown bold. CI=confidence interval, SEM=standard error of measurement; AP = anterior- posterior.

Mean (SD), along with reference values, were reported for these nine alignment variables and morphology measures with good reliability (Table 4). Significant differences in alignment and morphology between boys and girls were found for all the variables (*p* < 0.001), with girls having larger patellar tilt angle (16.0 vs. 12.2 degrees), larger bisect offset (67.1% vs. 63.8%), smaller epicondylar width (71.8 vs. 78.5 mm), higher medial and lateral condyle length to epicondyle width ratios (0.78 vs. 0.74 and 0.82 vs. 0.78, respectively), smaller patellar length (37.4 vs. 40.4 mm), smaller patellar width (36.99 vs. 39.42 mm), smaller femoral notch width (18.51 vs. 21.11 mm), and smaller femoral notch depth (22.7 vs. 23.1 mm) (Table 4).

**Table 4:** Mean (SD) and 2.5 and 97.5 centiles for the alignment variables and morphology measures with an concurrent validity ICC >0.75. values are shown for the total populations and for boys and girls separately.

Variable	Total study sample*			Boys ⁎⁎			Girls ⁎⁎⁎		
	*Mean (SD)*	*2.5 centile*	*97.5 centile*	*Mean (SD)*	*2.5 centile*	*97.5 centile*	*Mean (SD)*	*2.5 centile*	*97.5 centile*
Patellar tilt [degrees] a	14.2 (5.5)	4.2	25.5	12.2 (5.6)	3.2	25.5	16.0 (4.7)	7.5	25.4
Bisect offset [%]^b^	65.6 (11.3)	47.2	91.9	63.8 (10.6)	45.9	88.2	67.1 (11.5)	48.2	92.9
Epicondylar width [mm]^c^	74.8 (5.4)	65.6	85.9	78.5 (5.1)	67.7	87.9	71.8 (3.3)	65.2	79.2
Medial condyle length (AP) to epicondylar width ratio^c^	0.76 (0.04)	0.70	0.83	0.74 (0.03)	0.69	0.80	0.78 (0.03)	0.72	0.84
Lateral condyle length (AP) to epicondylar width ratio^c^	0.80 (0.03)	0.73	0.87	0.78 (0.03)	0.72	0.84	0.82 (0.03)	0.76	0.88
Patellar length [mm]^a^	38.8 (3.1)	33.0	45.2	40.4 (3.0)	34.6	46.4	37.4 (2.50)	32.4	42.1
Patellar width [mm]^a^	38.1 (3.2)	32.4	45.3	39.4 (3.4)	32.7	46.1	37.0 (2.5)	32.2	41.8
Femoral notch width [mm]^c^	19.7 (1.9)	16.5	23.5	21.1 (1.5)	18.4	24.1	18.5 (1.2)	16.3	21.1
Femoral notch depth [mm]^c^	22.9 (2.1)	18.7	27.0	23.1 (2.4)	18.1	27.6	22.7 (1.8)	19.2	26.2

All alignment variables and morphology measures differed significantly between boys and girls.

⁎ N = 3691 knees included in the total study sample,.

⁎⁎ N = 1755 knees from boys,.

⁎⁎⁎ N = 1936 knees from girls.

a n=53 missing,.

b n=389 missing,.

c n=336 missing.

Intra-rater ICCs between the twice manually annotated variables on MRI were all good or excellent (Appendix Table A1). The intra-rater ICCs in 3D were good or excellent for nine of the seventeen variables, and the concurrent validity between variables calculated using manually annotated landmarks on the 3D models and automatically extracted landmarks was good or excellent for ten of the seventeen variables (Appendix Tables A2 and A3).

## Discussion

Of the seventeen evaluated alignment variables and morphology measures, nine demonstrated good or excellent concurrent validity when comparing manual annotation on MRI with automatic extraction of landmarks from our SSM. Significant differences were found between boys and girls for all nine variables. In particular, girls showed a larger medial and lateral condyle length to epicondyle width ratio, indicating a narrower femoral width for the same length (AP) compared to boys.

Our primary analysis focused on comparing variables calculated using manually annotated landmarks on MRI (the gold standard) with those automatically extracted from 3D models. Data representation differed, as manual annotation on MRI was directly done on the image for acquiring 2D manual landmarks. The automatically extracted 3D landmarks were obtained from image-derived 3D models. Both differences in data representation (MRI vs. 3D models) and modes of analysis (manual vs. automatic) could influence the accuracy and clinical interpretations of the measurements. On one hand, differences between MRI and 3D models may stem from inherent differences such as the translation of MR imaging data into 3D volumetric data, which could affect the accuracy of localizing landmarks on 3D models. On the other hand, when using 3D models, we are not influenced by patient positioning during imaging, which can affect the accuracy of variables determined from MRI slices [22]. Furthermore, there are intra- and interrater differences in manual annotation, which could be minimized using automatic methods. However, there are also limitations to the automatic extraction of landmarks [23].

Nine out of the seventeen alignment variables and morphology measures demonstrated high ICCs when comparing automatic extraction to 2D manual annotation on MRI, the current gold standard, indicating good concurrent validity for these variables. The relatively low ICCs of many variables reflect the difference in annotation method and might not necessarily imply that 3D automatic extraction of landmarks is invalid. Instead, results may suggest that the 2D manual and 3D automatic landmark extraction do not measure the same. This is illustrated by two specific landmarks, *i.e*., the trochlear groove's deepest point (TGP) and the patella's most anterior point (PAP). In manual placement, the TGP landmark is positioned on the same slice as the anterior and posterior points of the femoral condyles, which is the slice showing the largest posterior femoral condyles. In 3D, this landmark can be located more distally or proximally, depending on the view. This influences the variables calculated using this specific landmark and, thus, the validity of the variables determined using automatically extracted landmarks compared to the current gold standard, *i.e*., manual annotation of the landmarks. In 3D annotation, the PAP may lie anywhere on the relatively flat anterior surface of the patella. While in the current manual annotation of MRI, the PAP is determined on the slice with the largest oblique length. These differences in landmark annotation between 3D space and 2D slices are reflected in the alignment variables and morphological measures calculated using these landmarks. For one of the variables using the PAP, patellar thickness, an attempt was made to standardize the calculation in 3D by using the sum of two projected distances to a plane instead of using the Euclidean distance between the two points itself (Fig. 2). However, this approach did not yield results consistent with manual annotations on 2D MRI slices. New variable definitions in 3D could be used to capture alignment variability and differences in morphology measures more accurately, but these must undergo thorough validation to ensure reliability and relevance.

We determined the intrarater reliability between manual annotation performed twice on MRI and on the 3D models. The intra-rater ICCs were all high for annotation on MRI. Intra-rater ICCs showed more discrepancies during manual annotation on 3D models, possibly because locating landmarks in 3D space is more challenging than identifying the same landmark in a single imaging plane. This is reflected in the higher SEM, higher bias, and wider limits of agreement for some alignment variables and morphology measures in 3D manual annotation compared to 2D annotation (Tables A1 and A2). Furthermore, we also determined the concurrent validity of manual annotation on 3D models to the automatic extraction of the landmarks from the 3D models (10 out of 17 ICCs>0.75). Although the outcomes are comparable, the 3D approach may be more biomechanically relevant, especially given that knee motion occurs in multiple planes simultaneously, and could be of additional value to clinical practice. The fact that almost half of the variables showed poor concurrent validity highlights the challenges of comparing landmarks annotated in 2D vs. those annotated in 3D. It underscores the need for clearly defined, accurate 3D variables that can more accurately capture real-world variations in knee bone shape.

The challenge of comparing 2D and 3D measures is not new. It has been explored before in the context of patellar instability [24] and orthopedic assessments, such as anterior cruciate ligament reconstruction (ACLR) or total knee arthroplasty (TKA) [22,25]. For example, posterior tibial slope, a key variable in ACLR and TKA, is typically measured on lateral radiographs. However, this method is susceptible to mispositioning, as rotational misalignment can easily introduce errors. In contrast, measurements using 3D imaging, such as CT, have shown more accurate and repeatable results [22]. While these improvements do not always rely on 3D models, they clearly illustrate how different imaging perspectives can substantially impact results. This emphasizes the importance of extracting landmarks in 3D, either directly from 3D imaging or from 3D models, to overcome the inherent limitations of 2D projections and obtain more reliable, biomechanically meaningful measures.

The magnitude of the significant differences identified between boys and girls for the nine alignment variables and morphology measures may be clinically relevant, particularly for patellar tilt (mean difference of 3.8 degrees), bisect offset (mean difference of 3%), and epicondylar width (mean difference of 6.7 mm). Especially since PFP patients are more often girls and higher patellar tilt and higher bisect offset ratios are more often reported [4].

Our bisect offset values (67% for girls and 64% for boys) differ considerably from those reported in the literature. Maine *et al*. [26] (2021) observed a mean bisect offset of 55% (SD = 6%) in adolescents [26]. While this discrepancy might suggest a systematic error, our Bland-Altman plots do not support this hypothesis, implying that the differences likely arise from the different annotation spaces (3D vs. 2D). For instance, the most anterior point of the medial femoral condyle does not necessarily corresponds with the slice that shows the widest width of the condyle, which is the slice used for manual annotation. Chen et al. [23] utilized automatic 3D annotations and reported bisect offset values similar to those of Maine *et al*. [26], albeit in an older study population with a mean age of 23 years. This may suggest an age-related morphological difference, although other factors, such as imaging modality (MRI vs. CT) and annotation protocols, may also play a role. The nine alignment variables that we determined showed good accuracy compared to the average of two manual annotations. Additionally, the intra-rater reliability of these manual annotations on MRI itself also showed high reliability. Therefore, we believe the values obtained in the present study are accurate, and the discrepancies with the literature likely result from differences in imaging modality or age differences.

### Strengths and limitations

A strength of our study is the large number of participants with available data at young adolescence. We demonstrated the feasibility of automated measurements in large study samples, which could, in future clinical settings, reduce reader time and variability. However, some limitations need to be addressed.

First, participant positioning during imaging was not rigorously standardized. All the scans were acquired with both legs in (near) full extension within the MRI coil, which could result in rotational misalignment or inconsistent knee flexion angles.

Second, our method of automated landmark extraction, based on a SSM, was not compared to other automated methods. While our SSM approach offers anatomical consistency [23], more accurate results might be obtained using other automated methods [27], or knowledge-based methods that utilize local shape information to identify edges, valleys, and sharp points [28].

The significant sex differences found between the nine alignment variables and the morphology measures might partly be attributed to differences in growth phase among the children included in the study. While there is no difference in age between the boys and girls who participated in the study, there is a difference in the timing of puberty onset and the growth phase. This was not included in this study, but data on this topic were published elsewhere [29].

While some of our ICCs demonstrated large confidence intervals, results remain valuable for future sample size calculations of validation studies. However, all manual intrarater ICCs were good to excellent, and we do not believe that a larger sample would have meaningfully altered the current findings.

Furthermore, interrater reliability was not formally assessed, although we did compare results between 3 different raters when developing the methods. Since interrater reliability may be more important in clinical settings, this should be formally evaluated prior to implementing these measures into clinical practice.

Lastly, many of the 3D alignment variables and morphology measures have been originally defined for 2D images. While 2D imaging discards valuable spatial information, simply applying traditional 2D-based definitions in 3D space may not suffice. To fully leverage 3D data, redefining alignment variables tailored for 3D analysis may be necessary. Future research should explore alternative approaches, including morphological landmark detection and the development of new 3D-specific clinical metrics.

## Conclusion

Several alignment variables and morphology measures can be accurately automatically determined in 3D, as demonstrated by nine automated measures of alignment and morphology, which showed good to excellent validity with significant differences observed between boys and girls. Our analysis advocates for a redefinition of key alignment variables and morphology measures to better suit anatomical analysis in 3D.

## Author contributions

Bierma-Zeinstra, van Middelkoop and Oei conceptualized the study. Van Paassen performed image segmentation under supervision of Hirvasniemi and Klein. Post-processing and statistical shape modeling were done by Van Paassen and Tumer under supervision of Zadpoor. Tumer automatically extracted all landmarks from the SSM. Bosch, Roos and Langius developed the method for automatic parameter calculation supervised by Van Paassen. Macri, Van Middelkoop and Bierma-Zeinstra supervised data analyzes. Piscaer and Macri supervised interpretation of the findings. Analysis and drafting the manuscript was done by Van Paassen under supervision of van Middelkoop. All authors provided input and detailed feedback on the manuscript during writing and everyone approved the final version that has been submitted.

## Funding sources

This work was supported by the Dutch Arthritis association (ReumaNederland, 21–1–204).

## Declaration of generative AI and AI-assisted technologies in the manuscript preparation process

During the preparation of this work, the author(s) used ChatGPT as extra help for debugging during the programming phase of the study. After using this tool, the author(s) reviewed and edited the content as needed and take(s) full responsibility for the content of the published article.

## Declaration of competing interests

The authors declare the following financial interests/personal relationships which may be considered as potential competing interests:

Marienke van Middelkoop reports financial support was provided by ReumaNederland. If there are other authors, they declare that they have no known competing financial interests or personal relationships that could have appeared to influence the work reported in this paper.

## Appendix Supplementary materials

Image, application 1
