# Supplementary material for: Automatic extraction of 2D patellofemoral bone morphology and alignment measures from 3D models in a young adolescent population
Source: Osteoarthr Imaging. 2026 May 12;6(3):100394. doi: 10.1016/j.ostima.2026.100394 (PMC13590919; doi:10.1016/j.ostima.2026.100394)
Supplement: Supplementary file 1 [file mmc1.docx]

**Appendix**

Figure A1: Bland-Altman plots of the remaining alignment variables and morphology measures with an ICC<0.75

Figure A2: Histograms of the nine alignment variables and morphology measures separate for boys and girls with the 2.5 and 97.5 Centiles shown in red dashes.

Table A1: Intrarater reliability between alignment variables and morphology measures calculated with twice manually annotation on MRI slices

| Alignment variable or morphology measure | ICC [95% CI] | SEM | Average (mean value) | Bias (mean difference) | Limits of agreement |
| --- | --- | --- | --- | --- | --- |
| Patellar tilt angle [degrees] | **0.98 [0.95; 0.99]** | 0.23 [0.16; 0.34] | 14.64 | -0.08 | -3.12; 2.96 |
| Lateral patellar tilt angle [degrees] | **0.94 [0.88; 0.97]** | 0.55 [0.38; 0.78] | 9.27 | -0.16 | -4.54**;** 4.23 |
| Lateral patellar translation [mm] | **0.85 [0.71; 0.93]** | 0.94 [0.66; 1.30] | 19.97 | -0.17 | -4.94; 4.60 |
| Bisect offset % | **0.99 [0.97; 0.99]** | 0.24 [0.17; 0.35] | 66.21 | 0.30 | -3.78; 4.39 |
| Sulcus angle [degrees] | **0.83 [0.67; 0.91]** | 1.52 [1.07; 2.11] | 143.12 | -0.30 | -7.44; 6.85 |
| Sulcus depth [mm] | **0.84 [0.68; 0.92]** | 0.25 [0.18; 0.35] | 5.43 | 0.04 | -1.19; 1.27 |
| Trochlear angle [degrees] | **0.82 [0.65; 0.91]** | 0.65 [0.46; 0.89] | 4.35 | 0.26 | -2.71; 3.23 |
| Inclination angle[degrees]  Lateral  Medial | **0.84 [0.69; 0.92]**  **0.84 [0.68; 0.92]** | 0.85 [0.60; 1.18]  1.04 [0.73; 1.44] | 18.88  162.00 | 0.26  -0.04 | -3.9; 4.42  -5.04; 4.97 |
| Epicondylar width [mm] | **0.99 [0.99; 1]** | 0.05 [0.04; 0.08] | 76.86 | 0.20 | -1.07; 1.47 |
| Condyle length (AP) to epicondylar width ratio  Lateral  Medial | **0.96 [0.92; 0.98]**  **0.96 [0.91; 0.98]** | 0.00 [0; 0]  0.00 [0; 0] | 0.79  0.78 | 0.00  0.00 | -0.03; 0.03  -0.03; 0.03 |
| Patellar [mm]  Length  Thickness  Width | **0.99 [0.97; 0.99]**  **0.86 [0.32; 0.96]**  **0.98 [0.97; 0.99]** | 0.07 [0.05; 0.10]  0.25 [0.14;0.55]  0.06 [0.04; 0.09] | 40.29  18.53  38.35 | 0.21  0.69  -0.02 | -0.95; 1.36  -0.62; 2.01  -0.97; 0.94 |
| Femoral notch width [mm] | **0.88 [0.76; 0.94]** | 0.39 [0.27; 0.55] | 20.02 | -0.19 | -2.36; 1.98 |
| Femoral notch depth [mm] | **0.79 [0.61; 0.89]** | 0.82 [0.58; 1.12] | 24.37 | -0.26 | -3.76; 3.24 |

*An ICC between 0.5 and 0.75 is deemed moderate, between 0.75 and 0.9 good and above 0.9 excellent. ICCs above 0.75 are shown bold. CI=confidence interval, SEM=standard error of measurement; AP = anterior- posterior.*

Table A2: Intrarater reliability between alignment variables and morphology measures calculated with twice manually annotated landmarks on 3D models

| Alignment variable or morphology measure | ICC [95% CI] | SEM | Average (mean value) | Bias (mean difference) | Limits of agreement |
| --- | --- | --- | --- | --- | --- |
| Patellar tilt angle [degrees] | **0.89 [0.78; 0.94]** | 0.96 [0.67; 1.34] | 14.92 | -0.56 | -6.16; 5.04 |
| Lateral patellar tilt angle [degrees] | 0.27 [-0.06; 0.55] | 5.99 [4.67; 7.20] | 15.53 | -3.13 | -16.86; 10.60 |
| Lateral patellar translation [mm] | 0.27 [-0.07; 0.56] | 3.12 [2.43; 3.76] | 11.79 | 1.34 | -5.80; 8.48 |
| Bisect offset % | **0.94 [0.89; 0.97]** | 0.75 [0.52; 1.07] | 64.56 | 0.15 | -6.11; 6.40 |
| Sulcus angle [degrees] | 0.48 [0.15; 0.71] | 3.45 [2.56; 4.42] | 155.01 | 0.57 | -8.81; 9.94 |
| Sulcus depth [mm] | 0.54 [0.23; 0.75] | 0.43 [0.32; 0.56] | 3.51 | -0.03 | -1.28; 1.22 |
| Trochlear angle [degrees] | 0.36 [0.00; 0.64] | 3.20 [2.41; 4.00] | 10.51 | 0.04 | -7.79; 7.88 |
| Inclination angle[degrees]  Lateral  Medial | 0.64 [0.38; 0.81]  0.25 [-0.13; 0.56] | 1.77 [1.28; 2.34]  5.87 [4.50; 7.19] | 16.36  164.39 | -0.57  -0.33 | -6.39; 5.24  -13.60; 12.95 |
| Epicondylar width [mm] | **0.98 [0.96; 0.99**] | 0.15 [0.11; 0.22] | 74.76 | -0.24 | -2.36; 1.88 |
| Condyle length (AP) to epicondylar width ratio  Lateral  Medial | **0.94 [0.88; 0.97]**  **0.96 [0.92; 0.98]** | 0.00 [0;0]  0.00 [0;0] | 0.79  0.77 | 0.00  0.00 | -0.03; 0.03  -0.03; 0.03 |
| Patellar [mm]  Length  Thickness  Width | **0.99 [0.98; 1.00]**  0.45 [0.12; 0.69]  **0.98 [0.97; 0.99]** | 0.03 [0.02;0.05]  1.49 [1.12; 1.87]  0.05 [0.04; 0.07] | 38.37  19.49  38.26 | -0.13  0.73  -0.09 | -0.80; 0.54  -3.19; 4.66  -0.89; 0.70 |
| Femoral notch width [mm] | **0.91 [0.82; 0.96]** | 0.25 [0.17; 0.35] | 20.79 | -0.26 | -1.90; 1,38 |
| Femoral notch depth [mm] | **0.90 [0.79; 0.95]** | 0.32 [0.22; 0.45] | 22.36 | 0.26 | -1.68; 2.20 |

*An ICC between 0.5 and 0.75 is deemed moderate, between 0.75 and 0.9 good and above 0.9 excellent. ICCs above 0.75 are shown bold. CI=confidence interval, SEM=standard error of measurement; AP = anterior- posterior.*

Table A3: Inter-method concurrent validity between manual annotation on 3D models and full automatic landmark extraction from the statistical shape mode and agreement between alignment variables calculated with manual annotated and automatically annotated landmarks.

| Alignment variable or morphology measure | ICC [95% CI] | SEM | Average (mean value) | Bias (mean difference) | Limits of agreement |
| --- | --- | --- | --- | --- | --- |
| Patellar tilt angle [degrees] | **0.90 [0.79; 0.95]** | 1.17 [0.80 ; 1.69] | 15.03 | -0.23 | -7.41; 6.94 |
| Lateral patellar tilt angle [degrees] | 0.17 [-0.4; 0.56] | 5.57 [4.08; 7.25] | 13.42 | 4.22 | -7.78; 16.22 |
| Lateral patellar translation [mm] | 0.66 [-0.12; 0.87] | 1.17 [0.71; 2.11] | 10.86 | 1.86 | -2.05; 5.78 |
| Bisect offset % | **0.91 [0.66; 0.97]** | 1.47 [0.90; 2.87] | 66.35 | -3.59 | -13.19; 6.00 |
| Sulcus angle [degrees] | 0.47 [-0.31; 0.81] | 2.82 [1.71; 4.44] | 157.96 | -5.91 | -13.51; 1.70 |
| Sulcus depth [mm] | 0.38 [-0.24; 0.76] | 0.43 [0.27; 0.61] | 3.00 | 1.02 | -0.05; 2.09 |
| Trochlear angle [degrees] | 0.32 [-0.31; 0.67] | 3.27 [2.3; 4.54] | 12.02 | -3.01 | -10.81; 4.78 |
| Inclination angle[degrees]  Lateral  Medial | 0.74 [0.46; 0.88] 0.33 [-0.42; 0.68] | 1.49 [1.03; 2.17]  5.47 [3.77; 7.95] | 16.26  163.92 | 0.19  0.94 | -5.58; 5.96  -12.15; 14.04 |
| Epicondylar width [mm] | **0.99 [0.99; 1]** | 0.06 [0.04; 0.08] | 74.65 | 0.20 | -1.36; 1.76 |
| Condyle length (AP) to epicondylar width ratio  Lateral  Medial | **0.97 [0.85; 0.99]**  **0.97 [0.93; 0.98]** | 0.00 [0;0]  0.00 [0;0] | 0.80  0.77 | -0.01  0.00 | -0.03; 0.01  -0.03; 0.03 |
| Patellar [mm]  Length  Thickness  Width | **0.99 [0.99; 1]**  **0.89 [0.76; 0.95]**  **0.97 [0.52; 0.99]** | 0.03 [0.02; 0.04]  0.34 [0.24; 0.49]  0.09 [0.05; 0.39] | 38.27  19.58  37.93 | 0.19  -0.17  0.66 | -0.50; 0.89  -2.16; 1.82  -0.36; 1.67 |
| Femoral notch width [mm] | **0.90 [-0.02; 0.97]** | 0.23 [0.12; 0.73] | 20.27 | 1.04 | -0.38; 2.46 |
| Femoral notch depth [mm] | **0.92 [0.67; 0.97]** | 0.29 [0.18; 0.58] | 22.73 | -0.75 | -2.73; 1.22 |

*An ICC between 0.5 and 0.75 is deemed moderate, between 0.75 and 0.9 good and above 0.9 excellent. ICCs above 0.75 are shown bold. CI=confidence interval, SEM=standard error of measurement; AP = anterior- posterior*

Table A4: Overview of the 17 alignment variables and morphology measures

| **Alignment variable or morphology measures** | **Variable/measure definition** | **ICC result (poor/fair/good/excellent)** | **Reference values provided (yes/no)** |
| --- | --- | --- | --- |
| Patellar tilt angle [degrees] | The angle between the line through the greatest width of the patella and the line through the posterior condyles | Good | Yes |
| Lateral patellar tilt angle [degrees] | The angle formed between the line drawn parallel to the lateral patellar facet and the line drawn connecting the most anterior points of the medial and lateral condyles | Moderate | No |
| Lateral patellar translation [mm] | Distance between two orthogonal lines on the posterior condyle line. One line through the deepest point of the trochlea and one line through the most posterior point of the patella | Poor | No |
| Bisect offset % | Percentage of the width of the patella that lies lateral to the line orthogonal to the posterior condyle line and through the trochlear groove | Excellent | Yes |
| Sulcus angle [degrees] | Angle between the lines from the medial anterior condyle and the lateral anterior condyle to the deepest point of the trochlear groove | Poor | No |
| Sulcus depth [mm] | Depth of the sulcus groove as compared to the anterior condyles | Poor | No |
| Trochlear angle [degrees] | The angle between the line through the anterior condyles and the line through the posterior condyles | Poor | No |
| Inclination angle[degrees]  Lateral  Medial | The angle between the lines through the deepest point of the trochlear groove and the most anterior point of the lateral condyle and the line connecting the posterior condyles  The angle between the lines through the deepest point of the trochlear groove and the most anterior point of the medial condyle and the lines connecting the posterior condyles | Moderate  Poor | No  No |
| Epicondylar width [mm] | Distance between the most medial and most lateral point of the femur | Excellent | Yes |
| Condyle length (AP) to epicondylar width ratio  Lateral  Medial | Ratio between lateral condyle length in the AP view and the epidondylar width  Ratio between the medial condyle length in the AP view and the epicondylar width | Excellent  Excellent | Yes  Yes |
| Patellar [mm]  Length  Thickness  Width | Distance between the most proximal and most distal point of the patella  2D distance between the most anterior and most posterior point of the patella  3D sum of the distance from the most anterior point to the plane defined by the most proximal, distal and *lateral/medial* point of the patella and the distance from the most posterior point of the patella to the same plane.  Distance between the most medial and most lateral point of the patella | Good  Moderate  Good | Yes  No  Yes |
| Femoral notch width [mm] | Inner width of the posterior femoral notch | Excellent | Yes |
| Femoral notch depth [mm] | Depth of the posterior femoral notch calculated as the distance of the deepest point of the femoral notch to the posterior condyle line | Good | Yes |
